# Obstructive fibrinous tracheal pseudomembrane in a cat

**DOI:** 10.1111/jvim.15944

**Published:** 2020-10-24

**Authors:** Ran Nivy, Ori Brenner, Vered Shub, Yaron Bruchim

**Affiliations:** ^1^ Koret School of Veterinary Medicine The Hebrew University of Jerusalem Rehovot Israel; ^2^ Department of Internal Medicine Ben‐Shemen Specialist Referral Center Ben‐Shemen Israel; ^3^ Department of Veterinary Resources Weizmann Institute Rehovot Israel

**Keywords:** distress, endotracheal tube, intubation, respiratory, trachea

## Abstract

Tracheal intubation (TI) is a common procedure that rarely entails life‐threatening complications. A 1.5‐year‐old female spayed cat presented with acute signs of respiratory distress 5 weeks after undergoing TI. Radiographs revealed a marked, segmental, tracheal narrowing. A hard, 5‐cm‐long, white‐yellowish tissue was identified and removed from the trachea, with subsequent resolution of clinical signs and radiographic changes. Microscopically, the tissue consisted of fibrin and lytic neutrophils, interspaced with optically empty cavities and a few remains of talcum powder and hair shafts. Consequently, a diagnosis of obstructive fibrinous tracheal pseudomembrane (OFTP) was made. A rare complication of TI in humans, OFTP should also be suspected in cats with respiratory distress, a history of TI and radiographic evidence of tracheal narrowing. Based on cases from other species and the cat described herein, the condition can be easily resolved with OFTP removal.

**Abbreviations**
BAL: bronchoalveolar lavage
ET: endotracheal tube
OFTP: obstructive fibrinous tracheal pseudomembrane
TI: tracheal intubation

## 1 INTRODUCTION

**FIGURE 1:**
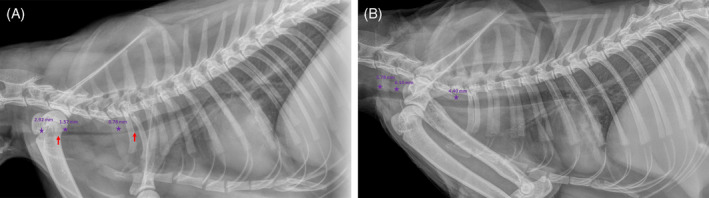
A, An approximately 4.8‐cm‐long, tracheal narrowing in a 1.5‐year‐old cat, beginning caudally to the cricoid and extending to the first rib, whereupon it abruptly ends (red arrows). A narrowing of the trachea can be appreciated throughout this segment (right and middle stars), which also extends, to a lesser degree, cranially (left star); B, immediately after the procedure, the narrowing was no longer appreciated with the removal of the obstructive fibrinous tracheal pseudomembrane

**FIGURE 2:**
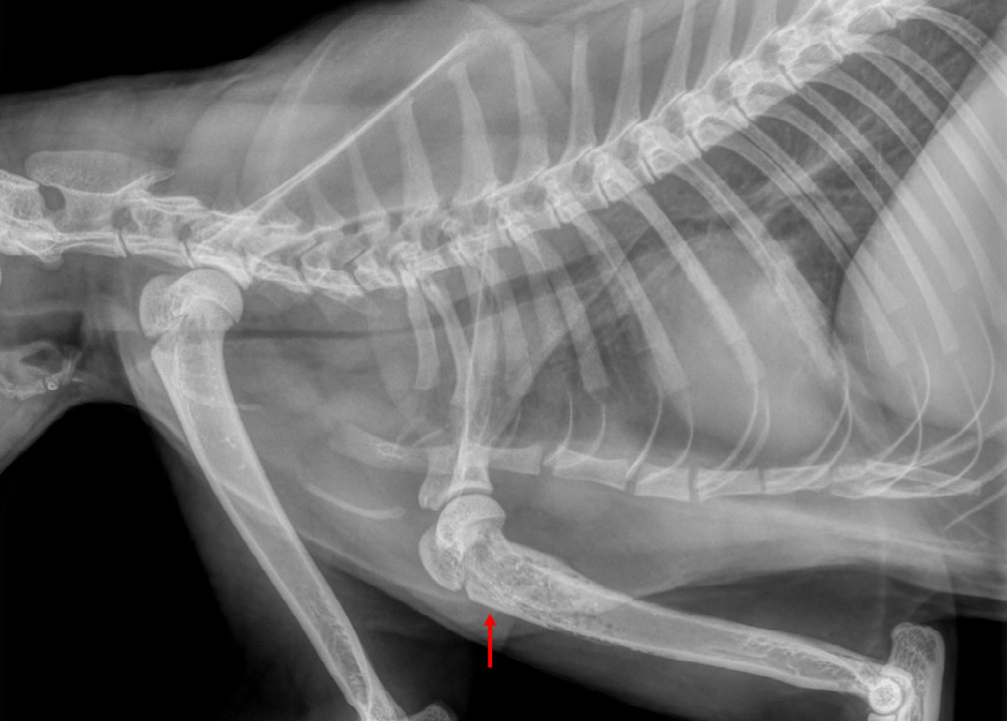
The right humeral proximal metaphysis has a moth‐eaten appearance with a tapering smooth periosteal reaction caudally. There is cortical thinning involving the proximal caudal metaphyseal bone as well as in the cranial diaphysis (red arrow). The left proximal metaphysis does not show similar changes and the cortical bone appears smooth on the periosteal and endosteal sides

**FIGURE 3:**
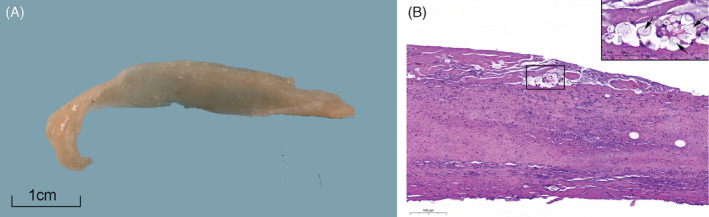
A, Grossly, the specimen consisted of a collection of white material of approximately 5 cm long, 1 cm wide, and 3 mm high. One aspect was slightly convex and the other slightly concave producing a shape that would conform to the inner wall of a circular structure such as the trachea. B, Microscopically, the material consisted of a mixture of fibrin and lytic neutrophils. Scattered throughout were multiple round to oval, optically empty cavities varying in size from approximately 20 to 300 μm in diameter. Rarely, these spaces contained strands of unidentified birefringent material or flocculent proteinaceous debris. A few remains of talcum powder and hair shafts were seen

A 1.5‐year‐old, female spayed, domestic shorthair cat presented to a referral center with a chief complaint of acute respiratory distress. Previous medical history included dermatophytosis and secondary bacterial pyoderma. Additionally, 5 weeks prior to presentation, the cat underwent general anesthesia, including tracheal intubation (TI), for the treatment of a suspected infected bite wound in her left hindlimb. Subsequently, the cat developed recurrent episodes of transient fever, which resolved and recurred over the course of 1 month, notwithstanding antibiotic treatment. The former included at first amoxicillin‐clavulonate (Augmentin, SmithKline Beecham, Brentford, UK; 10 mg/kg, PO, q12h) for 7 days, and then a combination of doxycycline (Doxylin, Dexcel Pharma, Or Akiva, Israel; 12.5 mg/kg, PO, q24h) and clindamycin (Clinacin, Chanelle Pharmaceuticals, Galway, Ireland; 12.5 mg/kg, PO, q12h), which the cat received until first presentation to the referral center. Medications were forcefully administered per os because the cat refused their voluntary consumption in the food. Upon presentation, abnormal physical examination findings included inspiratory and expiratory distress, with intermittent open‐mouth breathing and wheezes. Additional findings included skin lesions, which consisted of alopecia with crusted papules, affecting the convex aspect of both ear pinnae. Two smaller lesions were noted on the head and the dorsal aspect of the neck. Furthermore, the cat was lame on both the right front limb and the left hindlimb. Otherwise, the cat was alert and responsive, with a body condition score of 4/9, respiratory rate of 32 bpm, pulse of 160 bpm, and a rectal body temperature of 38°C. Subsequently, neck and chest radiographs were performed, and revealed a marked narrowing of the cervical trachea, beginning approximately at the level of the 4th cervical vertebra and extending to the first rib, whereupon the narrowing abruptly ended (Figure 1A). The narrowing of the trachea also extended cranially to the aforementioned segment, albeit to a lesser degree (Figure 1A). Additionally, radiographic changes consisting of a moth‐eaten appearance with a tapering, smooth periosteal reaction and cortical thinning were observed in both the proximal humerus and femur of the right frontlimb and left hindlimb, respectively (Figure 2). The cat was hospitalized with supplemental oxygen, butorphanol (Butomidor, Richter Pharma, Wels, Austria; 0.2 mg/kg, IV, q6h) and clindamycin (Rafa, Jerusalem, Israel; 12.5 mg/kg, IV, q12h). Because clinical signs had not resolved, and in an attempt to diagnose the cause for the respiratory distress, tracheal narrowing, and lameness, the cat was first anesthetized, intubated, and a blind, nonbronchoscopic bronchoalveolar lavage (BAL) was performed aseptically. The procedure entailed blind insertion of a sterile catheter through an endotracheal tube (ET) into the trachea until it no longer advanced, using 5 mL of sterile 0.9% saline fluid, for cytological and bacteriological evaluation. Therefore, the exact location of the catheter could not have been ascertained, and the aspirated fluid might have represented samples of primarily tracheal origin, rather than bronchoalveolar origin. Subsequently, a fluoroscopic‐guided, fine‐needle aspiration was performed from a lytic lesion in the right front limb, and an additional sample was procured for culture and sensitivity testing. Finally, ET was removed to perform tracheobronchoscopy. Attached to the ET was a hard, white‐yellowish tissue, which was approximately 5 cm long, 1 cm wide, and 3 mm high (Figure 3A). The tracheal mucosa was hyperemic with few superficial ulcerations in the cervical area and mild amount of purulent discharge, with no apparent narrowing, or collapse. The carina and major bronchial branches demonstrated no visual pathological changes.

The cat recovered and regained normal breathing in the absence of respiratory distress or abnormal breathing sounds. Additional X‐ray images revealed marked radiographic improvement in tracheal diameter immediately postprocedurally (Figure 1B). Cytological examination of BAL fluid demonstrated suppurative inflammation (>10 000 cells/μL; 85% neutrophils, 10% epithelial cells, and 5% mononuclear cells). Although bacteria had not been identified cytologically, bacterial culture revealed the presence of a resistant *Klebsiella pneumoniae* strain, which was only sensitive to amikacin and gentamicin, but not to amoxicillin/amoxicillin‐clavulonate, cephalexin, cefpodoxime, enrofloxacin, chloramphenicol, or trimethoprim‐sulfamethoxazole. Microscopic examination of the abnormal tracheal tissue demonstrated a mixture of fibrin and lytic neutrophils. Scattered throughout were multiple round to oval optically empty cavities varying in size from approximately 20 to 300 μm in diameter. Rarely, these spaces contained strands of unidentified birefringent material or flocculent proteinaceous debris. A few remains of talcum powder and hair shafts were seen (Figure 3B). The macroscopic and microscopic characteristics of this tissue were consistent with a diagnosis of obstructive fibrinous tracheal pseudomembrane (OFTP).

Cytological evaluation of the FNA sample from the right front limb was unremarkable, with no evidence of fungal or mycobacterial organisms (a Ziehl‐Neelsen staining was not performed, however), and no evidence of neoplasia. The same bacterial species (ie, *K pneumoniae*) was also isolated from the lesion in the right front limb, albeit of a different strain with a different antibiotic sensitivity profile when compared to the resistant strain obtained from the BAL fluid (this strain was susceptible to most antibiotic families tested).

Twenty‐four hours after the procedure the cat was discharged from the hospital without any signs of respiratory distress as described upon presentation. Clindamycin and marbofloxacin (Marbocyl, Vetoquinol, Lure Cedex France; 5 mg/kg, PO, q24h) were administered at home, in addition to meloxicam (Loxicom, Norbrook, Newry, North Ireland; 0.1 mg/kg, PO, q24h, for 3 days). Respiratory clinical signs had not recurred during the following year. However, the cat experienced at least 2 recurrent fever episodes, for which further diagnosis was not pursued by the referring veterinarian. Furthermore, the outcome of the osseous lesions was similarly unclear since X‐ray studies had not been repeated.

## 2 DISCUSSION

Obstructive fibrinous tracheal pseudomembrane characteristically consists of a collection of fibrin, interspaced with leukocytes and desquamated necrotic epithelial cells. It is essentially a desiccated mucus plug that forms circumferentially in the trachea, without adhering to the adjacent tracheal wall.^1^ It has previously been reported in humans^1^, ^2^, ^3^ and in 1 dog,^4^ but has yet to be described in cats. In humans, OFTP formation invariably follows TI, with the exception of a single case report of a silicone stent‐induced OFTP.^5^ Clinical signs referable to OFTP often appear shortly after extubation, with a median time to clinical signs of 24 hours (interquartile range, 6‐96 hours).^2^ However, clinical signs can appear as early as 1 hour postextubation and infrequently can be delayed for up to 15 days.^2^, ^3^, ^6^ Duration of intubation is similarly variable, ranging from 1 hour to 17 days (mean, 113 hours) in 1 review^3^ and has not been linked to increased likelihood of OFTP development. Clinical signs commonly include stridor, unrelenting cough, and in the more severe cases respiratory distress and failure from tracheal obstruction and resultant hypoxemia and hypercapnia^1^, ^2^, ^3^, ^5^, ^6^, ^7^ Nevertheless, the valve‐like manner of the intratracheal OFTM can result in intermittent, positional breathing difficulties.^1^ Moreover, human patients who are too exhausted might not be able to generate stridor, and rarely, cases where the OFTP has not detached from the tracheal wall can remain asymptomatic.^1^, ^6^ In the present case, the cat exhibited intermittent episodes of respiratory distress, which had transiently resolved, especially with sedation, and then recurred, presumably the result of positional changes of the pseudomembrane. Additionally, the occurrence of a milder tracheal narrowing cranial to the level of the 4th cervical vertebra, the presumed cranial termination of the OFTP, and the resolution of this narrowing after removal of the OFTP might have resulted from concurrent tracheal collapse consequent to increased negative pressures during respiration. Thus, intermittent tracheal collapse might also have contributed to intermittent episodes of respiratory distress.

Obstructive fibrinous tracheal pseudomembrane can be suspected based on compatible history and clinical signs after extubation, corroborated by neck X‐rays and ascertained during tracheobronchoscopy. Nonetheless, this complication has only recently received attention in human medicine and is still believed to go unrecognized in many cases.^1^ The latter might account for a small number of OFTP‐related deaths,^1^, ^2^ notwithstanding the easily treatable nature of the disease.

The pathogenesis of OFTP remains poorly understood. Its formation likely stems from superficial damage to the tracheal wall since subepithelial stroma is often absent in histology, and deep macroscopic lesions are not appreciated during tracheobronchoscopy after its removal.^2^, ^3^, ^6^ A common hypothesis associates increased ET‐cuff pressure with tracheal wall injury and subsequent development of OFTP, analogous to other ET‐associated complications including tracheal stenosis.^2^, ^3^ However, in many cases, ET pressure was monitored and was deemed appropriate, some cases were intubated for less than 1 hour, while some were intubated with low‐pressure ET or cuffless tubes, thus partially refuting the cuff‐induced ischemic injury theory.^2^, ^3^, ^7^ Similarly, bacteriological and fungal cultures have repeatedly failed to reveal an underlying infectious etiology to the disease, although in a small subset of human patients an underlying infectious respiratory disease might have precipitated the formation of OFTP.^3^ Acidic aspiration of gastric content and consequent localized tracheal damage is yet another theory that has not been corroborated.^2^ In the present case, the instigating trigger remains unknown, notwithstanding several possible etiologies. Firstly, the cat had undergone ET intubation 5 weeks prior to presentation. In humans and in 1 case report in a dog,^4^, ^6^ clinical signs often present shortly after extubation, although rarely can present as long as 2 weeks postprocedurally.^6^ The present case is therefore unique in the unusually protracted time interval until clinical signs appeared. The presence of talcum, which often originates from gloves, and hair shafts, a common iatrogenic contaminant of histological preparations, in the OFTP is also suggestive of ET‐associated origin for its formation, linking the surgical procedure the cat had underwent with the development of the OFTP. In this scenario, it is presumed that clinical signs appeared only 5 weeks postprocedurally secondarily to sudden detachment of the pseudomembrane from the tracheal wall and tracheal obstruction. Secondly, the cat was forcefully administered 2 antibiotics, which had previously been associated with esophageal stricture, and of which doxycycline in particular is known to cause esophageal mucosal irritation.^8^, ^9^ In theory, tracheal aspiration of antibiotics, and specifically doxycycline, might have caused tracheal mucosal irritation and subsequently OFTP formation, but the effect of doxycycline on tracheal mucosa has not been investigated and remains speculative. Thirdly, the presence of bacteria is believed to be secondary to the OFTP and impaired clearing of tracheobronchial secretions, but alternatively might have caused tracheal inflammation and OFTP formation. The latter is supported by the presence of lytic neutrophils in histology, notwithstanding the absence of bacteria. In humans, bacterial and fungal tracheitis can produce a viscous, adherent, tracheal membrane, but its clinical and histological features markedly differ from those of OFTP,^10^ rendering an infectious etiology an unlikely cause of OFTP formation.

Regardless of its cause, OFTP, if recognized early, is easily treatable and rarely recurs. Furthermore, spontaneous expectoration thereof has anecdotally been reported in humans.^11^ The single case report of a dog with OFTP was unusual owing to its recurring nature.^4^ In the present case, as in humans, clinical signs immediately resolved after removal of the OFTP and have not recurred during the following 1 year.

In conclusion, OFTP, hitherto unreported in cats, should be suspected in cases of respiratory distress, associated with a history of TI and radiographic evidence of tracheal narrowing. Based on cases from other species and the cat described herein, the condition can be easily resolved with removal of the obstructive tissue and rarely recurs.

## 3 CONFLICT OF INTEREST DECLARATION

Authors declare no conflict of interest.

## OFF‐LABEL ANTIMICROBIAL DECLARATION

Authors declare no off‐label use of antimicrobials.

## INSTITUTIONAL ANIMAL CARE AND USE COMMITTEE (IACUC) OR OTHER APPROVAL DECLARATION

Authors declare no IACUC or other approval was needed.

## HUMAN ETHICS APPROVAL DECLARATION

Authors declare human ethics approval was not needed for this study.
